# Images in Medicine: Recognizing Hereditary Hemorrhagic Telangiectasia Through Mucocutaneous Findings and Its Management Challenges

**DOI:** 10.7759/cureus.108121

**Published:** 2026-05-01

**Authors:** Stephanie Khodzandi, Muhammad Rajib Hossain, Evan Moritz, Mahzabin Kibria, Anna Mccomas

**Affiliations:** 1 Internal Medicine, Jamaica Hospital Medical Center, New York, USA; 2 Primary Care, Efficient Medical & Dental Care PC, New York, USA; 3 Medicine, Sir Salimullah Medical College, Dhaka, BGD

**Keywords:** arteriovenous malformations, autosomal dominant disorder, congenital vascular disorder, hereditary hemorrhagic telangiectasia (hht), mucocutaneous telangiectasia, osler-weber-rendu syndrome, recurrent epistaxis

## Abstract

Hereditary hemorrhagic telangiectasia, or Osler-Weber-Rendu syndrome, is a rare autosomal dominant vascular disorder characterized by dysregulated angiogenesis due to abnormalities in the transforming growth factor-β (TGF-β)/bone morphogenetic protein (BMP) signaling pathway that result in mucocutaneous telangiectasias and visceral arteriovenous malformations, leading to recurrent bleeding and multisystem involvement. This image-based case highlights characteristic mucocutaneous telangiectasias and illustrates the challenges of long-term disease management, including recurrent hospitalizations and limited durability of vascular interventions. The case emphasizes the importance of coordinated outpatient care and consideration of targeted therapies to reduce morbidity and bleeding recurrence.

## Introduction

Hereditary hemorrhagic telangiectasia (HHT), also known as Osler-Weber-Rendu syndrome, is an autosomal dominant vascular dysplasia characterized by mucocutaneous telangiectasias and visceral arteriovenous malformations (AVMs). The estimated prevalence ranges from approximately one in 5,000 to one in 8,000 individuals worldwide, though the condition is likely underdiagnosed due to variable clinical expression [1,2].

HHT results from pathogenic variants in genes encoding proteins involved in the transforming growth factor-β (TGF-β)/bone morphogenetic protein (BMP) signaling pathway. Up to 90% of disease-causal variants are observed in ENG (HHT type 1) and ACVRL1 (HHT type 2), with SMAD4 and GDF2 (also known as BMP9) less frequently responsible for HHT [1]. SMAD4 variants account for approximately 2-3% of HHT cases and are associated with a combined syndrome of juvenile polyposis (JP) and HHT (JP-HHT overlap syndrome), which occurs in 15-81% of patients with germline SMAD4 pathogenic variants [3-5]. GDF2 variants, while rare, have been identified in patients meeting diagnostic criteria for HHT and are sometimes referred to as HHT type 5 [6,7]. Diagnosis is established clinically using the Curaçao criteria, which include spontaneous recurrent epistaxis, mucocutaneous telangiectasias at characteristic sites, visceral AVMs, and a first-degree relative with HHT [2,8]. Fulfilling ≥3 of these criteria is sufficient for a definitive diagnosis.

Epistaxis is the most common manifestation, occurring in more than 95% of patients with HHT, with an average age of onset of approximately 12 years, and 90% developing epistaxis before the age of 30 years [9-11]. This frequently leads to chronic iron-deficiency anemia and impaired quality of life [1,12]. Mucocutaneous telangiectasias typically involve the lips, oral cavity, tongue, face, hands, and nail beds, increasing in number with age [1]. Recognition of these characteristic findings is essential, as early diagnosis enables screening for pulmonary, cerebral, and hepatic AVMs, which carry risk of stroke, brain abscess, high-output cardiac failure, and other complications [1,11]. According to the Second International HHT Guidelines, screening for visceral or cerebral AVMs can be done with the use of Doppler ultrasonography, transthoracic echocardiogram with contrast, and MRI [2]. Patients with SMAD4 variants require specialized surveillance for both HHT-related vascular lesions and gastrointestinal surveillance for juvenile polyps, with screening recommended within the first six months of life or at diagnosis due to a higher risk of gastric polyposis and gastrointestinal cancer compared to other HHT subtypes [4,5,13].

This case serves as an image-based learning opportunity demonstrating classic mucocutaneous manifestations of advanced HHT and highlights the management challenges associated with transfusion-dependent disease.

## Case presentation

**Figure 1:**
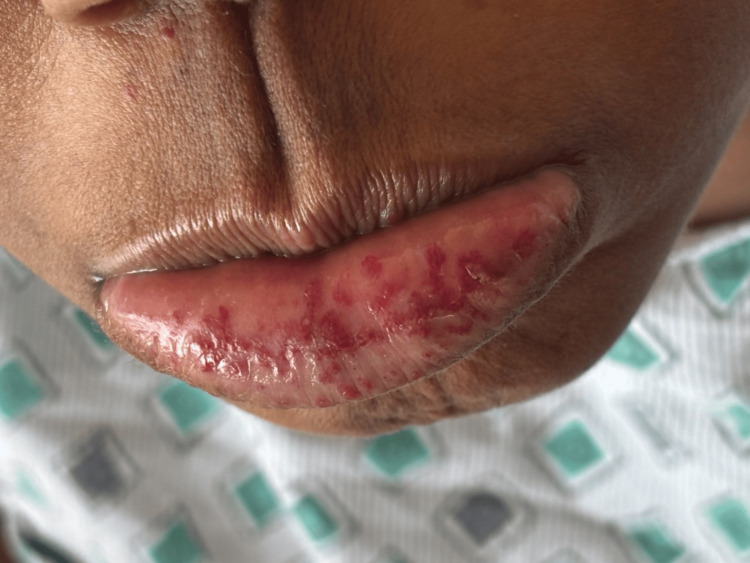
Telangiectasias of the lower lip appearing as multiple red-to-violaceous punctate lesions consistent with mucocutaneous involvement of hereditary hemorrhagic telangiectasia (HHT). These fragile, superficial dilated vessels are characteristic of HHT and are a major source of recurrent bleeding.

**Figure 2:**
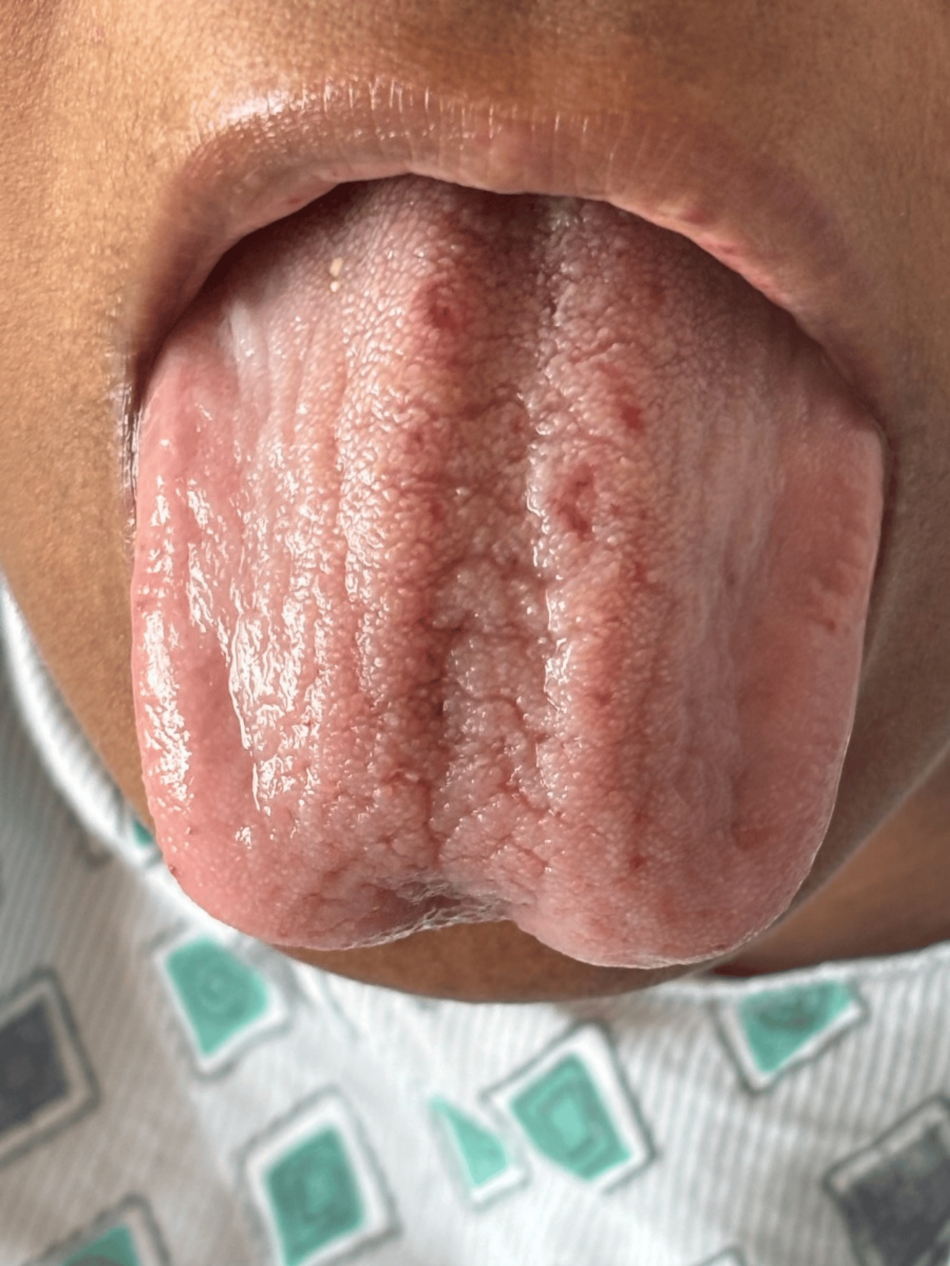
Prominent telangiectasias involving the tongue, a common site of mucosal vascular lesions in hereditary hemorrhagic telangiectasia, and a contributor to chronic blood loss.

**Figure 3:**
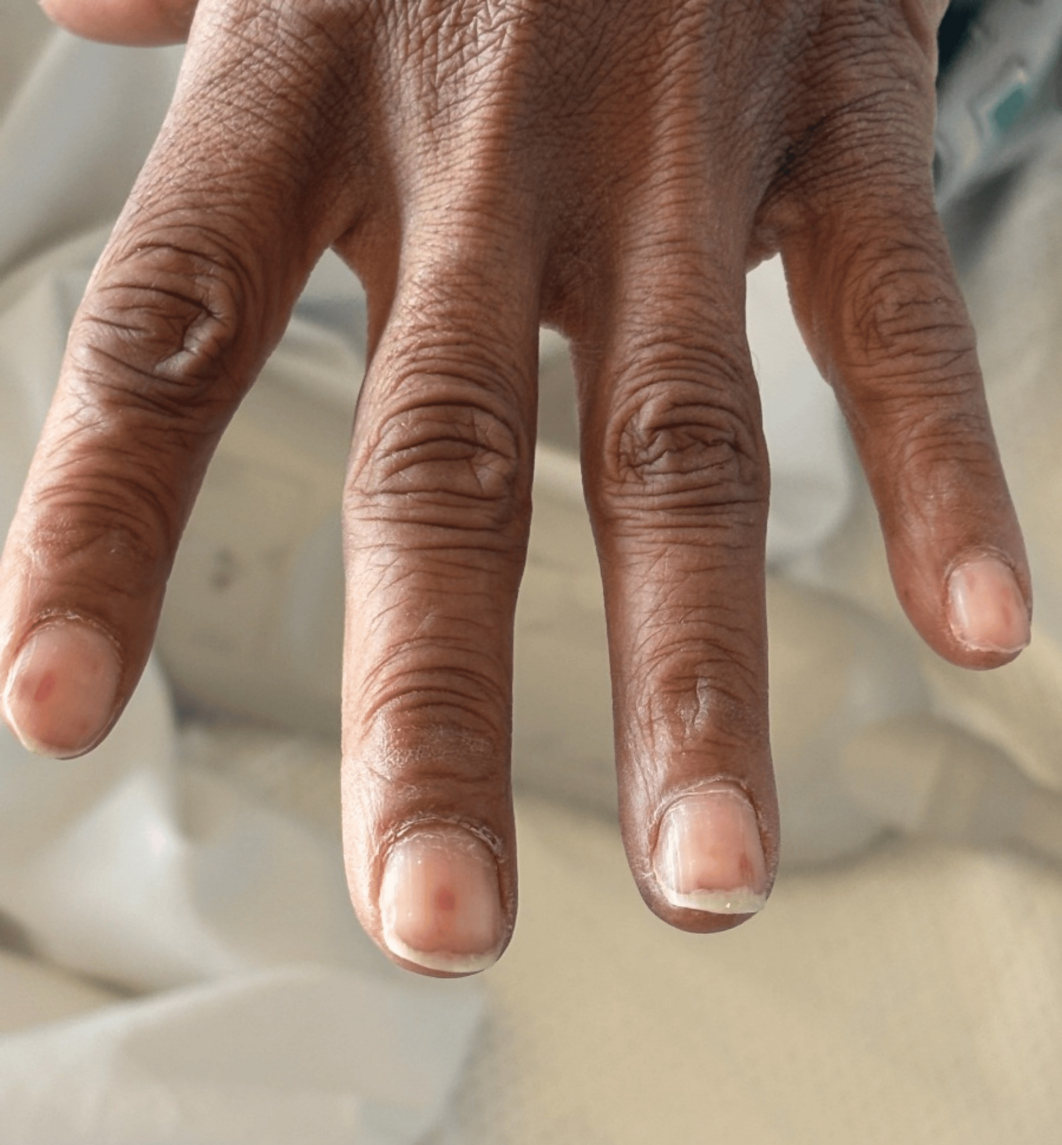
Nail bed telangiectasias demonstrating dilated superficial blood vessels, characteristic of systemic vascular involvement in hereditary hemorrhagic telangiectasia.

**Figure 4:**
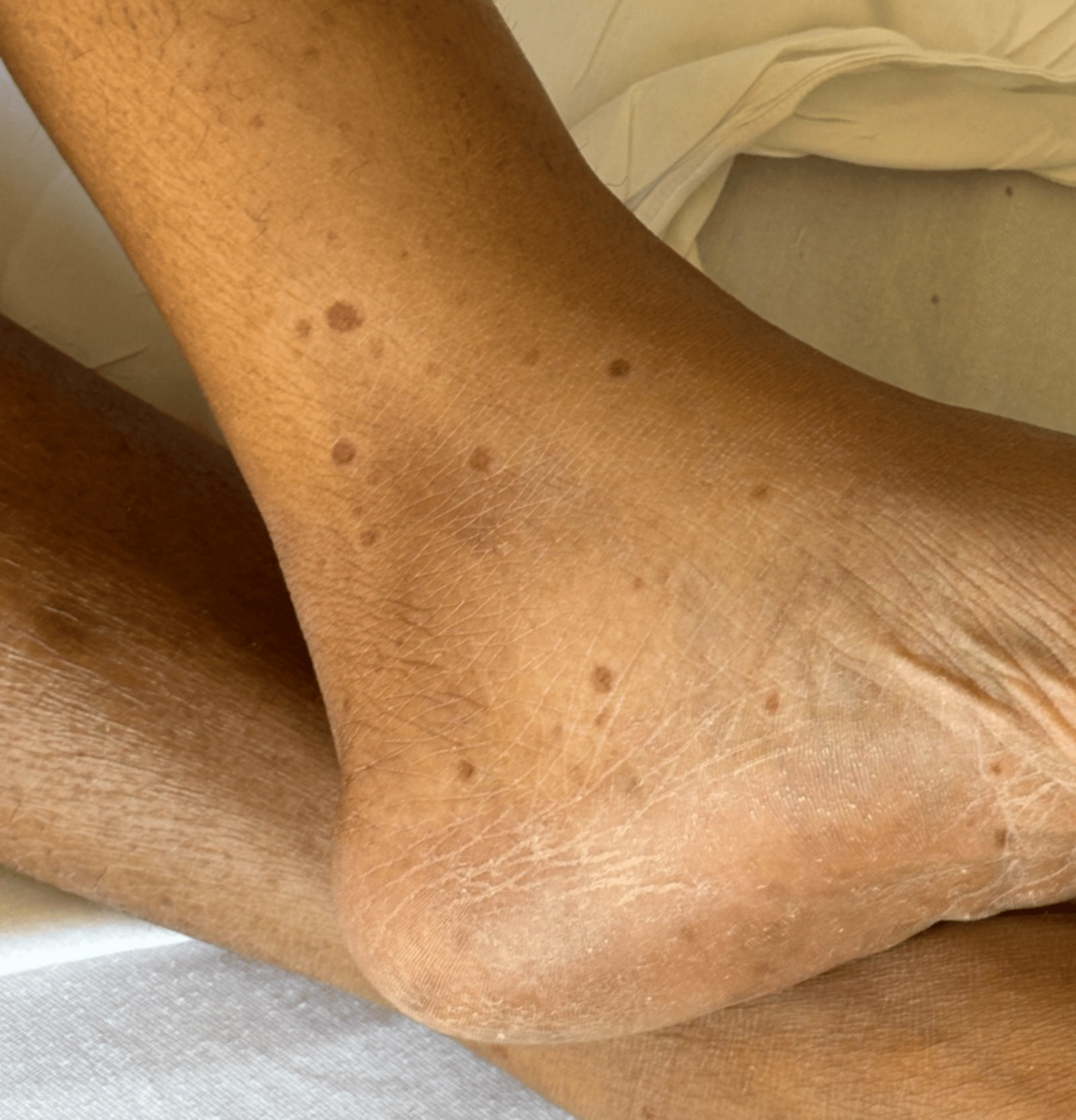
Telangiectasias in the leg appearing as purple spots.

A 39-year-old female with HHT presented with recurrent severe epistaxis and transfusion-dependent chronic anemia, resulting in multiple hospital admissions for blood transfusions. The patient had a known family history of HHT; however, she had not undergone any genetic testing. She was asymptomatic for most of her life and became severely symptomatic after her last pregnancy in 2020. Clinical photographs revealed numerous mucocutaneous telangiectasias involving the lips, tongue, oral mucosa, nail beds, and skin (Figures 1-4). These superficial dilated vessels were prone to spontaneous rupture and chronic blood loss. On screening evaluations, prior brain MRIs have shown evidence of small cavernous malformations. CT of the abdomen and pelvis done in 2024 demonstrated multiple small hepatic AVMs, while recent CT angiograms of the chest revealed no pulmonary AVMs. Based on these findings, the patient fulfilled all four previously described Curaçao criteria, supporting a definitive diagnosis of HHT.

The patient’s clinical course had been complicated by frequent hospitalizations for severe recurrent epistaxis, chronic anemia requiring repeated transfusions, and pulmonary hypertension due to HHT. She also suffered from end-stage renal disease, requiring hemodialysis and congestive heart failure, both of which developed secondary to comorbid conditions, limiting her eligibility for the use of systemic antifibrinolytic and anti-angiogenic therapies. In 2023, she underwent bilateral sphenopalatine artery embolization, which resulted in only transient improvement. Ongoing bleeding despite embolization underscores the limited durability of emergency vascular interventions in advanced HHT and contributes to significant healthcare resource utilization.

## Discussion

There is no curative therapy for HHT, and management is directed toward reducing bleeding, preventing complications, and screening for visceral AVMs [1,2]. Epistaxis represents the most prevalent and clinically burdensome manifestation. Studies report that approximately one-third of patients develop chronic anemia, and a subset progress to transfusion dependence, particularly in advanced disease [12]. Our patient's recurrent, severe epistaxis and transfusion dependence align with reported high-morbidity phenotypes.

First-line management strategies for epistaxis include humidification, topical emollients, avoidance of nasal trauma, and iron supplementation [1,2]. When conservative measures fail, targeted endonasal interventions such as laser photocoagulation, septodermoplasty, and sclerotherapy may reduce the severity of bleeding [2,14]. Sclerotherapy has been shown to significantly reduce nasal bleeding and the need for nasal packing immediately following the procedure [15,16]. However, published series demonstrate variable durability, with many patients requiring repeat procedures due to the progressive and diffuse nature of mucosal telangiectasias [14]. Arterial embolization, including sphenopalatine artery embolization performed in this patient, is typically reserved for severe or refractory epistaxis. Literature suggests that embolization often provides only temporary control, as collateral vessel formation and persistent telangiectatic fragility contribute to recurrent bleeding [17,18]. The transient improvement observed in this case, followed by recurrent severe epistaxis, is consistent with these findings, highlighting the limited long-term durability of emergent vascular interventions in advanced HHT.

Systemic therapies have gained attention in patients with refractory bleeding. Antifibrinolytics such as tranexamic acid have demonstrated significant reductions in epistaxis severity scores in randomized studies [19,20]. More recently, anti-angiogenic therapy with intravenous bevacizumab has shown promising reductions in transfusion requirements and improvement in high-output cardiac failure in select severe cases [21,22]. However, systemic therapy requires careful patient selection and monitoring due to risks including hypertension, thrombosis, and impaired wound healing [21]. In patients such as ours, with congestive heart failure and end-stage renal disease, therapeutic decisions must carefully balance bleeding control with systemic risk.

Additionally, expert consensus guidelines emphasize multidisciplinary management through coordinated care involving hematology, otolaryngology, and primary care providers [1,2]. Recurrent hospitalizations for transfusion, as seen in this case, reflect a systems-level challenge frequently reported in severe HHT populations. Transitioning stable patients to structured outpatient transfusion programs and longitudinal specialty follow-up may reduce inpatient utilization while improving continuity of care.

Overall, this case reflects a severe, advanced phenotype of HHT characterized by extensive mucocutaneous involvement and transfusion-dependent anemia. The patient's clinical course parallels published data demonstrating the chronic, relapsing nature of HHT-related epistaxis and underscores the need for integrated, multidisciplinary management strategies beyond episodic emergency intervention.

## Conclusions

This case illustrates a common management and systems-based challenge in HHT: recurrent inpatient admissions for transfusion-dependent bleeding. The extent of mucocutaneous telangiectasias often corresponds with disease severity and bleeding burden, emphasizing the importance of thorough physical examination, early recognition, and prompt systematic screening for visceral involvement. With consistent follow-up and coordinated care through a primary care physician or hematologist, many patients may be managed more effectively in an outpatient transfusion center, potentially reducing hospital admissions while improving continuity of care.
